# Comparative analysis between 2D and 3D colorectal cancer culture models for insights into cellular morphological and transcriptomic variations

**DOI:** 10.1038/s41598-023-45144-w

**Published:** 2023-10-26

**Authors:** Zaid Nsaif Abbas, Ali Z. Al-Saffar, Saba Mahdi Jasim, Ghassan M. Sulaiman

**Affiliations:** 1 Department of Molecular and Medical Biotechnology, College of Biotechnology, Al-Nahrain University, Jadriya, Baghdad, Iraq; 2 Oncology Teaching Hospital, Medical City, Ministry of Health, Baghdad, Iraq; 3 Division of Biotechnology, Department of Applied Sciences, University of Technology, Baghdad, Iraq

**Keywords:** Cancer, Cell biology, Drug discovery, Molecular medicine, Oncology

## Abstract

Drug development is a time-consuming and expensive process, given the low success rate of clinical trials. Now, anticancer drug developments have shifted to three-dimensional (3D) models which are more likely to mimic tumor behavior compared to traditional two-dimensional (2D) cultures. A comparative study among different aspects was conducted between 2D and 3D cultures using colorectal cancer (CRC) cell lines, in addition, Formalin-Fixed Paraffin-Embedded (FFPE) block samples of patients with CRC were used for evaluation. Compared to the 2D culture, cells grown in 3D displayed significant (*p* < 0.01) differences in the pattern of cell proliferation over time, cell death phase profile, expression of tumorgenicity-related genes, and responsiveness to 5-fluorouracil, cisplatin, and doxorubicin. Epigenetically, 3D cultures and FFPE shared the same methylation pattern and microRNA expression, while 2D cells showed elevation in methylation rate and altered microRNA expression. Lastly, transcriptomic study depending on RNA sequencing and thorough bioinformatic analyses showed significant (*p-adj* < 0.05) dissimilarity in gene expression profile between 2D and 3D cultures involving thousands of genes (up/down-regulated) of multiple pathways for each cell line. Taken together, the study provides insights into variations in cellular morphologies between cells cultured in 2D and 3D models.

## Introduction

The process of drug development is time-intensive and cost-effective, in which several phases start from target identification, discovery and optimization, pre-clinical validation conducting in vitro and in vivo experiments, and clinical trials to drug approval for clinical use^1^. Approximately, 90% of discovered drugs that reached the clinical trial phase failed to make it to FDA certification and commercialization^2^. Hence, such a low success rate in clinical trials dramatically affects the process of drug development making it a slow and costly business. For this reason, an imperative need for alternative and invented technologies that can minimize the risk of drug development failure. Two of the most promising areas anticipated to enhance the success rate of drug development are the advancement of new biomarkers and the availability of new pre-clinical models that significantly mimic in vivo biology^3^.

Cell-based in vitro assays are simple, rapid, and inexpensive, as well as versatile and easily reproducible compared to expensive animal models^4^. To date, mainly two-dimensional (2D) cell culture techniques have been used in drug discovery^5^. However, 2D cultures do not necessarily reflect the complex microenvironment that cells located in a tissue. One of the major influences that have a great impact on our understanding of the limited physiological applicability of 2D cultures is the interactions between cells and their surrounding extracellular matrix (ECM). The ECM is not only characterized by its biochemical composition but also by its physical and mechanical properties important for maintaining homeostasis^6^. The structure of the ECM and its physical properties can affect a cell's response to drugs by either improving drug efficacy, altering a drug's mechanism of action, or promoting drug resistance^7,8^.

Recently, cancer drug development has switched to a 3D culture model which emerged as an advanced research tool to simulate tumor behavior and characteristics more than traditional 2D models^9^. Resemble tumors, 3D cultures accommodate surface-lying and deeply buried cells, proliferating and non-proliferating regions, oxygen-deprived hypoxic cells, and a well-oxygenated outer layer of cells^10^. Such 3D models have been successfully used in screening environments to identify potential cancer therapeutics^11^. The ability of in vitro 3D culture systems to produce uniform spheroids of human cancer cells, combined with the ability to study spheroid response at a high growth rate and on a large scale, represents an important step toward facilitating more relevant and accurate testing^12^.

The tumor cell line can be utilized in different approaches including primary monolayers, complex 3D systems, and xenograft models. A major leap in the use of these cells came with the introduction of 3D cell culture. The technique of 3D culture is now the most preferred approach for applying tumor cell lines to fill the gap between “absolute in vitro” and “true in vivo”^13,14^. Aspects of cancer biology to which 3D cell culture practices have contributed and provided include tumor cell morphology, cellular microenvironment, gene expression, metastasis (invasion and migration), angiogenesis, metabolism, drug discovery, chemotherapeutic assays, adaptive responses, and cancer stem cell applications^15,16^. Concerning 2D cell culture which is still a valuable application for cell-based research, nevertheless, might provide unpredicted and misleading data on in vivo responses^17^.

Precision medicine, also known as personalized medicine, tailor’s disease prevention and treatment to individual variations in genes, environments, and lifestyles^18^. Its goal is precise treatment for the right patients at the right time^19^. Currently, the location of each gene in the reference genome is well known, and precise mutations can be identified and isolated, allowing specific diseases to be targeted at the genetic level^20^. The transcriptome, a snapshot of cell states, reveals gene function, genome plasticity, and expression regulation^21,22^, making it valuable for studying dynamic cancer cells^19^. The transcriptome represents all transcripts in a cell at a specific stage or condition, crucial for understanding genome functionality, cell components, and disease^23,24^.

The genes that are turned on or off in a cell, their transcription levels, and the timings at which they activate can all be determined using RNA-seq^25^. This enables researchers to comprehend a cell's biology and assess alterations that might point to a disease^26^. Transcriptional profiling, single nucleotide polymorphism (SNP) discovery, RNA editing, and differential gene expression analysis are some of the most well-liked RNA-seq methods^27,28^.

As mentioned earlier, this study aimed to prove the validity of two null hypotheses. The first assumes that there is no significant difference between 2D and 3D cell culture techniques. The second hypothesis states that the 3D culture technique is closest to cell growth in vivo in terms of architecture, proliferation, and behavior. To answer these questions, we performed several experiments utilizing five different colon cancer cell lines as well as colon cancer tissues in terms of 2D/3D technique differences at morphological, epigenetic, and molecular levels.

## Materials and methods

### Cell lines, tissue samples and therapeutics

Five different human colorectal adenocarcinoma cell lines (Caco-2, HCT-116, LS174T, SW-480 and HCT-8) were kindly provided by MonoJo Biotech Company, Amman–Jordan and were used in this study. All colorectal cell lines were routinely cultured in Dulbecco's modified eagle medium with HEPES (DMEM; Biowast, France) supplemented with 10% fetal bovine serum (FBS; Biowast, France) and 1% of Glutamine-Penicillin-Streptomycin 100× solution (Biowast, France). The cells were maintained in culture flasks (25 cm^2^, ThermoScientific, USA) under a humidified atmosphere of 5% CO_2_ at 37 °C. After reaching 80–90% confluency, cells were harvested using 2–3 mL of trypsin–EDTA (0.025%) solution to detach cells for subculturing. For spheroidal 3D cultures, an aliquot of 200 µL of cell suspension (5 × 10^3^ cells) was added into individual wells of Nunclon^TM^Sphera super-low attachment U-bottom 96-well microplates (ThermoScientific, USA). Spheroids were maintained in a complete medium (37 °C, 5% CO_2_, humidified) with three consecutive 75% medium changes every 24 h.

Formalin-fixed paraffin-embedded (FFPE) frozen (− 80 °C) surgical resection samples (*n* = 50) of former patients suffering from colorectal cancer (CRC) were collected from Oncology Center, Baghdad Teaching Hospital, Medical City, Baghdad-Iraq under relevant guidelines and regulations of the declaration and regulation of Helsinki in 1975 as principles of ethical statement. Permissions were obtained from the Oncology Center and approved by the institutional ethical committee of Al-Nahrain University, College of Biotechnology (Ref. No. COB 3857/2020). Informed consent and/or assent were obtained from the participants. A 15–20 µm sections were sliced and used for DNA and RNA extraction. Inclusion criteria included FFPE samples of colon adenocarcinoma (Ascending, transverse, descending colon to the level of rectosigmoid junction) and FFPE blocks should be not older than 2 years. Samples with a history of preoperative radiotherapy, rectal tumors, other tumors, recurrent CRC, and inadequate FFPE for DNA and RNA extraction were excluded from the study. Doxorubicin (Doxo) and cisplatin were purchased from Merck (USA), while 5FU (5-Flurouracil) was obtained from Sandoz (Austria).

### Cell proliferation assay

The proliferation rate in 2D and 3D cultures was compared using colorimetric CellTiter 96® Aqueous Non-Radioactive Cell Proliferation Assay Kit (Promega, USA). In brief, CRC cells were cultured at 2D and 3D conditions and at an initial cell concentration of 5 × 10^3^ cells/well. At the desired incubation period, 20 µL of tetrazolium/phenazine methosulfate mixture (MTS/PMS, 20:1 v/v) was pipetted into each well of assay plate containing 100 µL of culture. The plate was incubated for 4 h at 37 °C. The bio-reduction of MTS into soluble formazan by metabolically active cells was detected at 490 nm absorbance using an ELISA plate reader (Bio-Rad, USA)^29^.

### Cellular apoptosis analysis

In this experiment, the apoptotic/live status of tumor cells cultured in 2D and 3D was compared using FITC Annexin V Apoptosis Detection Kit I (BD Biosciences, USA). After incubation (24 h for 2D cultures and 72 h for 3D cultures), cells were harvested using gentle trypsinization and washed twice with ice-cold Hanks Balanced Salt Solution (HBSS) and collected by centrifugation for 10 min at 1200 rpm at ambient temperature. Cells then resuspended to a final concentration of 1 × 10^6^ cells mL^-1^ in Annexin-binding buffer. Cells (in 100 µL) were stained simultaneously with 5 µL of FITC-labeled Annexin V and 5 µL of propidium iodide (PI) for 15 min at room temperature. An aliquot of 400 µL of binding buffer was added and mixed gently. Cells were analyzed with a fluorescence-activated cell flow cytometer (FACSCalibur Flow Cytometer, BD) and data were analyzed with FacsDiva software (Version 5.0.3). Four cell populations were distinguished: live cells (Annexin negative and PI negative), early apoptotic cells (Annexin positive and PI negative), late apoptotic (Annexin positive and PI positive), and dead cells (PI positive)^30^.

### RNA extraction and purification

Total RNA was extracted from CRC cell lines and FFPE samples according to the protocol of the TRIzol™ Reagent Kit (ThermoFisher, USA) as instructed by the manufacturer. Cells were directly lysed by adding 0.1 mL TRIzol™ reagent and the lysate was homogenized by pipetting several times. For FFPE samples, a set of three to four 10 µm tissue sections from each FFPE block were collected in 1.5 mL tubes and incubated at 56 °C for 15 min to soften the paraffin wax, followed by deparaffinization in xylene and 100% ethanol^31^. For each tube, 0.6 mL of TRIzol™ reagent was added to lyse tissue. DNase I was performed to purify RNA. Purified RNA samples were stored at − 30 °C for downstream application. Quantus™ fluorometer (Promega, USA) was used to detect the concentration and the purity of extracted RNA. The percentage of RNA fragments above 200 nucleotides (DV_200_) and RNA integrity number (RIN) for FFPE blocks were determined using the 2100 Bioanalyzer System (Agilent Technologies, USA).

### Reverse transcriptase qPCR (RT-qPCR) experiment

RT-qPCR was used to measure gene expression at the transcription level for *CD44*, *ANXA1*, *KTR18*, *SOX2*, and *OCT4* genes. cDNA was synthesized from 1 µg total RNA using GoScript™ Reverse Transcription System (Promega, USA) and RT-qPCR was performed using GoTaq qPCR Master Mix (Promega, USA). The relative quantification of gene expression was achieved by normalization with the housekeeping *GAPDH* gene as control and calculated by the comparative threshold cycle (Ct) method. Matured small RNA sequences (*miR-144-5p*, *miR-146a-5p*, *miR-155-5p,* and *miR-21-5p*) were downloaded from the miRNA database site (https://www.mirbase.org/) and specific stem-loop RT-qPCR primers were designed using the miRNA Design Tool and U6 snRNA as reference gene. Primer sequences used in this experiment are listed in Table 1.

**Table 1:** The sequence of primers used in this study.

Gene	Sequence 5′-3′	Annealing Tem. (°C)	References
*CD44* (F)	AAGGTGGAGCAAACACAACC	60	^32^
*CD44* (R)	AGCTTTTTCTTCTGCCCACA	60	
*ANXA1* (F)	GATTCAGATGCCAGGGCCT	60	^33^
*ANXA1* (R)	CACTCTGCGAAGTTGTGGAT	60	
*KTR18* (F)	GTGGTGCTCTCCTCAATC	60	^33^
*KTR18* (R)	GCTCTGGGTTGACCGTGG	60	
*SOX2* (F)	CCCAGCAGACTTCACATGT	60	^33^
*SOX2* (R)	CCTCCCATTTCCCTCGTTTT	60	
*OCT4* (F)	CCTCACTTCACTGCACTGTA	60	^34^
*OCT4* (R)	CAGGTTTTTCTTTCCCTAGCT	60	
*GAPDH* (F)	TCTCCTCTGACTTCAACAGCGAC	60	^35^
*GAPDH* (R)	CCCTGTTGCTGTAGCCAAATTC	60	
*miR-144-5p* (RT)	GTTGGCTCTGGTGCAGGGTCCGAGG	60	Designed by Authors
	TATTCGCACCAGAGCCAACCTTACA		
*miR-144-5p* (F)	GTTGGGGGATATCATCATATAC	55	Designed by Authors
*miR-146a-5p* (RT)	GTTGGCTCTGGTGCAGGGTCCGAGG	42	
	TATTCGCACCAGAGCCAACAACCCA		
*miR-146a-5p* (F)	GTTTGGTGAGAACTGAATTCCA	55	Designed by Authors
*miR-155-5p* (RT)	GTTGGCTCTGGTGCAGGGTCCGAGG	42	
	TATTCGCACCAGAGCCAACACCCCT		
*miR-155-5p* (F)	GTGGGTTAATGCTAATCGTGAT	55	Designed by Authors
*miR-21-5p* (RT)	GTTGGCTCTGGTGCAGGGTCCGAGG	42	
	TATTCGCACCAGAGCCAACTCAACA		
*miR-21-5p*	GTTTGGTAGCTTATCAGACTGA	55	Designed by Authors
U6 snRNA (RT)	GTTGGCTCTGGTGCAGGGTCCGAGG	55	
	TATTCGCACCAGAGCCAACAAAATA		
U6 snRNA (F)	TTGGTGCTCGCTTCGGCA		
Universe miRNA	GTGCAGGGTCCGAGGT		

R: Reverse; F: Forward; RT: Reverse Transcriptase; Temp: Temperature.

### LDH cytotoxicity assay

The viability of CRC cell lines cultured in 2D and 3D models after exposure to Doxo, 5FU and cisplatin was investigated using CyQUANT™ Lactate Dehydrogenase (LDH) cytotoxicity Assay Kit (ThermoFisher, USA). CRC cells (1 × 10^5^ cells/well) were cultured in 2D model for 24 h and in 3D model for 72 h. Both cultures were incubated at 37 °C in the presence of 5% CO_2_. The culture medium was discarded, and 200 µL of fresh culture medium containing serial dilutions of Doxo, 5FU, and cisplatin with a concentration range of 16.6, 33.3, 62.5, 125, 250 and 500 µg mL^-1^. Plates were incubated at 37 °C for 24 h (2D) and 72 h (3D). Following drug exposure, the media was discarded. Then 50 µL of stop solution to each well was added and absorbance was measured at 490 nm and 680 nm, simultaneously. LDH activity was determined by subtracting the 680 nm absorbance value (background) from the 490 nm absorbance and cytotoxicity was calculated using the following formula^36^.

$$\mathrm{Cytotoxicity \%}=\left(\frac{\mathrm{Compound Treated LDH Activity}-\mathrm{Spontaneous LDH Activity}}{\mathrm{Maximum LDH Activity}-\mathrm{Spontaneous LDH Activity}}\right)\times 100$$

All treatments were run in triplicates and IC_50_ values were calculated by linear approximation regression of the percentage survival versus the drug concentration.

### Detection of global DNA methylation

#### Genomic DNA extraction

Genomic DNA was isolated from CRC cell lines cultured in 2D and 3D models as well as FFPE samples according to the protocol of ReliaPrep™ Blood gDNA Miniprep System (Promega, USA).

#### DNA methylation assay

Colorimetric assay, MethylFlash™ Methylated DNA Quantification Kit (EpiGenetik, USA) was used to quantify 5-methylcytosine (5-mC) level in CRC cell lines cultured in 2D and 3D models as well as FFPE samples. Based on kit protocol, genomic DNA samples were diluted to reach a concentration of 5 ng µL^−1^ and 80 µL of the binding solution was added to 20 µL DNA in each well and the plate was incubated at 37 °C for 90 min. After washing 5-mC antibody was added and the reaction was completed by adding a stop solution. Changing the color to yellow was measured using a microplate reader at 450 nm. The absolute amount of methylated DNA was calculated by generating a standard curve, and optical density (OD) values were plotted versus the amount of positive control (ME3) at each concentration point. Finally, the slope (OD/ng) was determined using a linear regression equation according to the following formula.

$$-mC (ng)=\frac{\mathrm{Sample OD}-ME3 OD}{\mathrm{Slope }\times 2}$$

### RNA sequencing and data mining

RNA library preparation and sequencing for CRC cell lines cultured in 2D and 3D models as well as FFPE samples were performed using TruSeq™ Stranded Total RNA with Ribo-Zero Kit (Illumina, USA). Briefly, poly-A RNA was purified from total RNA (200 ng) using oligo(dT). Abundant rRNA was removed from purified total RNA using the Ribo-Zero Plus rRNA Depletion Kit followed by fragmenting and priming the depleted RNA using random hexamers for cDNA synthesis. Agencourt AMPure XP beads were used to clean up the RNA library. The library was put into a flow cell for cluster production, where fragments are captured on a lawn of surface-bound oligos that are complementary to the library adapters. Using bridge amplification, each fragment was multiplied into unique, clonal clusters. After cluster creation was finished, NovaSeq 6000 was used to sequence the templates (Illumine, USA) with 150 bp long of reading and 100× coverage raw data.

Data related to RNA Sequencing were analyzed by the Galaxy platform^37^ with the following pipeline: Reads were trimmed using the Trimmomatic algorithm^38^, while Quality Scores were assessed using the FastQC algorithm^39^. Reads were aligned to the *Homo sapiens* genome build hg38 using the HISAT2 algorithm^40^. Individual sample reads were quantified using the FeatureCounts algorithm^41^ and normalized via Relative Log Expression (RLE) using the EdgeR algorithm^42^. Moreover, fold changes, p-values, and an optional covariate correction were calculated using FeatureCounts. Partitioning Around Medoids (PAM) approach was used to group genes for the final heatmap of differentially expressed genes^43^. For the enrichment analysis, several database sources were used, including Interpro, NCBI, MSigDB, REACTOME, and WikiPathways.

### Statistical analysis

Statistical analyses were performed using GraphPad Prism version 9.0 (GraphPad Software Inc., La Jolla, CA). The paired *t*-test, one-way, and two-way ANOVA were performed to determine the statistically significant differences between various groups. The data were presented as mean ± SD of the mean, and statistical differences were defined as * *p* < 0.05 or ** *p* < 0.01. All experiments were at least achieved in triplicates.

## Results

### Cell culture and cell proliferation

**Figure 1:**
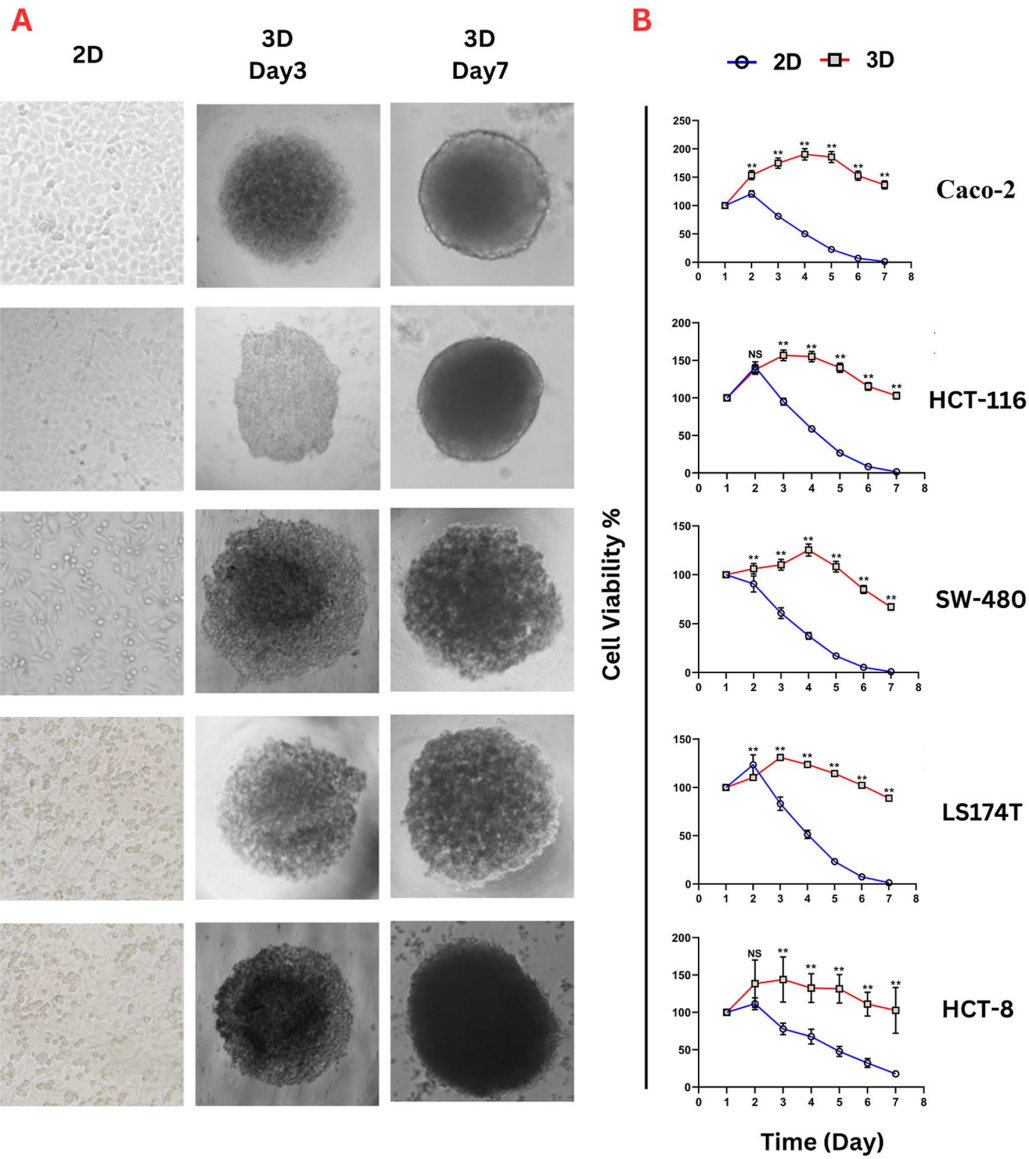
**(A)** Morphological growth characteristics of colorectal-derived tumor spheroids. Caco-2, HCT-116, SW-480, LS147T, and HCT-8 cell lines were grown in 3D U-bottom 96-well plates for seven days and compared with cells grown in the 2D system. Images of 3D cultures were captured by inverted microscopy on day 3 and 7 (Scale bar = 200 µm), and 2D images (day 2) were captured using an inverted microscope (40 × magnification). **(B)** Cell Titer 96® AQueous One Solution Cell Proliferation assay for CRC cell lines growing in 2D and 3D cell culture systems at 37ºC, 5% CO_2_. Mean cell viability (%) was detected for 7 days and normalized with day one (initial seeding). ** *p* < 0.01, NS: Non-Significant (*n* = 6).

Cell proliferation of CRC cell lines was monitored in both 2D and 3D models, while cell viability and stability were compared between both models. In 3D cell culture, for all cell lines, cells started to clump and adhere to adjacent cells after the first hours of seeding, increasing cell density and mass within the subsequent days. An initial cell count (6000 cells/100 µL) was used as an optimum number as a preliminary test to determine the optimal number of starting cells. Figure 1A, shows the growth development stages and cell mass formation of Caco-2, HCT-116, SW-480, LS147T, and HCT-8 cells within seven days of incubation. Cells exhibited the capability to form spheroids. However, morphological differences were observed after day 3 of incubation among different cell lines, while on day seven, all cell types aggregated into spheroids. Furthermore, all CRC cell lines observed no migration from spheroids to the medium at the end of the experiment. Morphologically, Caco-2 and HCT-116 displayed a smooth surface, while the spheroids of other cell lines showed rough surfaces. The cells of all tested cell lines that were maintained on 2D cultures showed confluency (˃80%) on day 2 incubation (Fig. 1A).

When comparing the pattern of cell proliferation between two types of cell culture (2D and 3D) using CRC cell lines, the results showed a significant variation in cell proliferation between 2 and 3D culture techniques (Fig. 1B, Supp. 1). Regarding the 2D cultures, the cells showed the highest proliferation rate after 24 h of incubation, with a subsequent decline in cell viability following day three. This proliferation pattern in the 2D system was observed in all CRC cell lines without changing media. Compared to the 2D system, cells grown in the 3D system displayed significant (*p* < 0.01) differences in the pattern of cell proliferation over time. For Caco-2 and SW-480 cell lines, the 3D cell culture technique showed a maximum proliferation rate (190.6 ± 10.01 and 125.4 ± 6.2%, respectively) on day 4 of incubation. The stability in cell viability revealed a slow rate of decline in cell proliferation with ˃70% cell maintenance after seven days of incubation than the traditional 2D during the first three days.

The maximum proliferation rate in 3D cultures for HCT-116, LS174T, and HCT-8 (156.7 ± 7.0, 131.03 ± 3.2, and 143.9 ± 30.2%, respectively) was noted on day 3 of incubation. The viability of cells grown in 3D culture lasted until day 7, maintaining the slow decrease in cell proliferation rate at the minimum for all cell lines without daily changing media. On the contrary, the growth rate of cells in a 2D culture lasted for three days, followed by a significant regression in the growth rate compared with 3D cultures.

### Cellular apoptosis profile in 2D and 3D cultures

**Figure 2:**
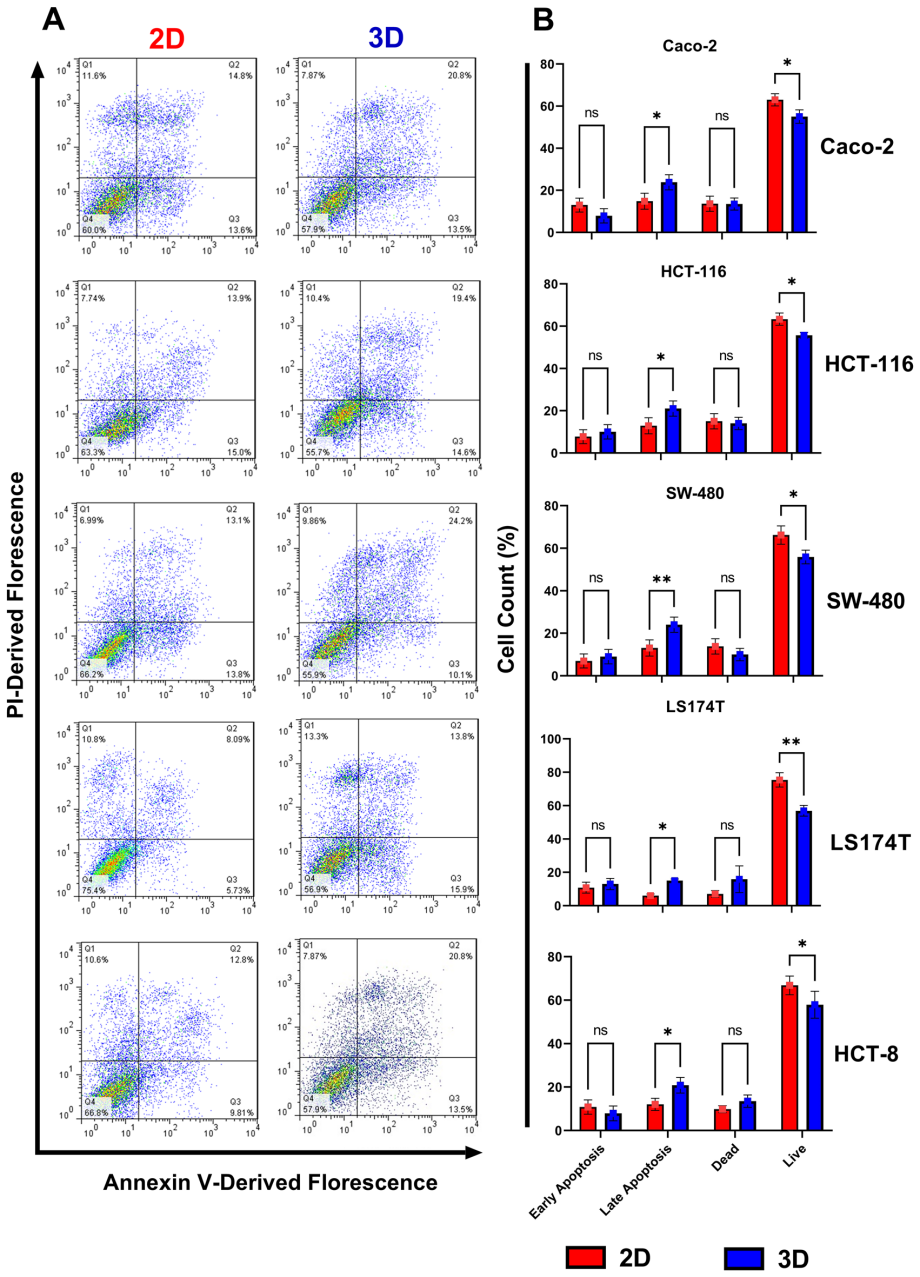
**(A)** Annexin V-FITC/PI staining to evaluate apoptosis in Caco-2, HCT-116, SW-480, LS174T, and HCT-8 cell lines were grown in 2D (24 h) and 3D (72 h) models. Cell phase status was determined using flow cytometry measuring Annexin-FITC labeling and PI uptake by the cell. Three independent experiments were conducted, and FacsDiva 5.0.3 software was used to analyze data. **(B)** Cell population count percentage of Caco-2, HCT-116, SW-480, LS174T, and HCT-8 cell lines grown in 2D and 3D cell culture. Histograms correspond to cell count distribution of live, dead, and apoptotic cells in early and late stages. Data represents mean ± standard deviation of three independent experiments. * *p* < 0.05; ** *p* < 0.01; NS: Non-significant; FITC: Fluorescein Isothiocyanate; PI: Propidium Iodide; 2D: Two- Dimensional; 3D: Three- Dimensional.

The cell death phase (live, dead, and apoptotic cells) of CRC cell lines cultured in 2D, and 3D models were investigated using FITC-labeled Annexin/PI staining (Fig. 2A). Flow cytometric results of cell lines cultured in 2D conditions for 24 h and 3D conditions for 72 h showed no distinction in the count of early apoptotic and necrotic cells. In contrast, the rate of late apoptotic cells in 3D cell culture was significantly higher than cells maintained in 2D culture (*p* = 0.0184, 0.0269, 0.0066, 0.0472, and 0.0418 for Caco-2, HCT-116, SW-480, LS174T and HCT-8, respectively). Such elevation was accompanied by a significant decline in the live-cell population grown in 3D culture conditions compared to the cells grown in monolayer culture (*p* = 0.0392, 0.0399, 0.0102, 0.0001 and 0.0391 for Caco-2, HCT-116, SW-480, LS174T and HCT-8, respectively) (Fig. 2B).

### Expression of *ANXA1*, *CD44*, *KRT18*, *OCT4*, and *SOX2OT* Genes

**Figure 3:**
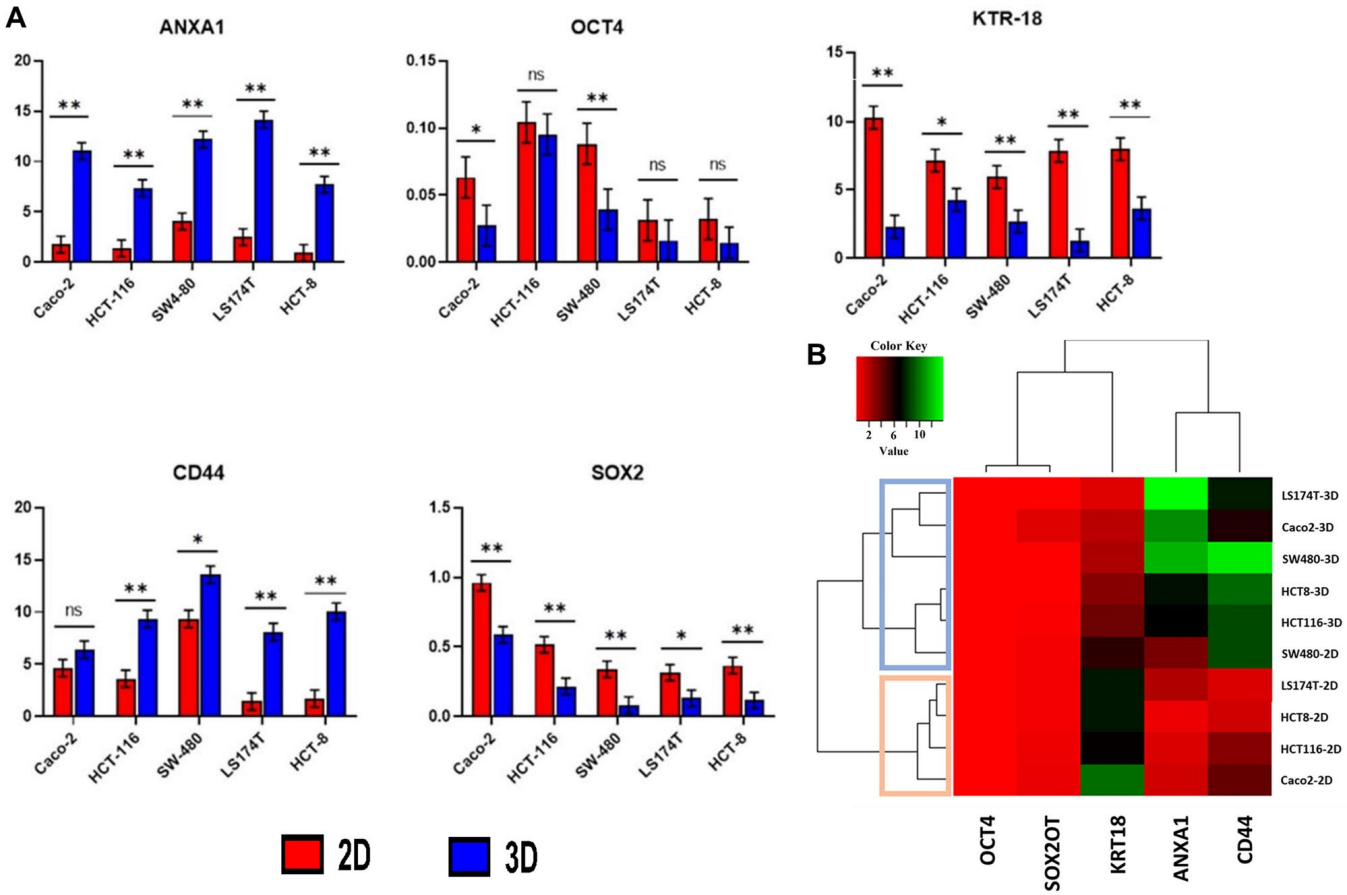
**(A)** RT-qPCR analysis comparing gene expression of *ANXA1*, *CD44*, *KRT18*, *OCT4*, and *SOX2OT* genes in CRC cell lines across different culturing techniques. Data represented as mean ± standard deviation of the normalized fold change of three independent experiments. * *p* < 0.05; ** *p* < 0.01; NS: Non-significant; 2D: Two- Dimensional; 3D: Three- Dimensional. **(B)** Gene expression analysis of CRC cell lines grown in 2D and 3D culture conditions. Sample data were pooled and normalized to compute the cluster dendrogram. Heat map representing gene at two experimental conditions clustered based on expression values (used heatmapgenerator5 tool).

By comparing 2D and 3D cell culture techniques regarding the expression of *ANXA1*, *CD44*, *KRT18*, *OCT4*, and *SOX2OT* genes, folding results represented variation in gene expression between tumor cells cultivated in 2D and 3D cultures. Figure 3A,B highlights the significant up-regulation of *ANXA1* (*p* = 0.0011) and *CD44* (*p* = 0.0087) genes in their expression with 3D cell culture spheroids compared with 2D monolayer cells in all tumor cell lines (except for Caco-2, no significant differences appeared in *CD44* expression). Paradoxically, *KRT18* and *SOX2OT* showed significantly decreased expression in 3D cultures compared with the 2D model (*p* = 0.0071 and *p* = 0.0005, respectively). On the other hand, the behavior of *OCT4* expression was diverse among different cell lines, in which no variation in expression was detected in HCT-116, LS147T, and HCT-8 cell lines between 2 and 3D models, while significantly down-regulated in Caco-2 and SW-480 cells cultivated in 3D culture compared with the 2D model (*p* = 0.036 and *p* = 0.0034, respectively). The gene expression of *ANXA1*, *CD44*, *KRT18*, *OCT4*, and *SOX2OT* genes was further completed from data extracted from RNA sequencing analysis (Supp. 2). Based on RNA sequencing analysis, the pattern of *ANXA1*, *CD44*, *KRT18*, *OCT4*, and *SOX2OT* gene expression was highly similar to that obtained from RT-qPCR results, confirming the differences CRC cell lines cultured in 2D and 3D models.

### Cellular response to anticancer drugs

The response of colon tumor cells to the anticancer drugs Doxo, 5FU and cisplatin were studied in 2D and 3D cell culture techniques using LDH cytotoxicity assay. The cytotoxicity results (IC_50_) for treated cells showed a significant difference (*p* < 0.01) in cellular response toward anticancer drugs between 2D and 3D cultures. Data analysis showed that the most effective drugs needed higher concentrations to reach IC_50_ in the case of 3D cell culture compared to the same treatments in the case of 2D cultures. Generally, all cells grown in 3D tumor spheroids showed significantly (*p* < 0.01) less sensitivity to increasing concentrations of chemotherapeutic agents than in 2D cultures. 5-FU was significantly (*p* < 0.01) more potent in killing CRC cells grown in 2D systems compared to those cultured in 3D-based spheroids system. All spheroidal tumor cells in 3D cultures revealed a significant (*p* < 0.01) higher resistance to cisplatin treatments than in 2D cultures. Moreover, the efficiency of Doxo in reducing the viability of the tested tumor cell line was significantly (*p* < 0.01) low in 3D spheroidal cell cultures compared to 2D cultures. Variation in dose–response curves was distinctly observed between 2D and 3D cultures concerning all chemotherapeutic treatments, indicating differences in the cellular response to Doxo, 5FU, and cisplatin in 3D cultures compared to cells maintained in the 2D system (Supp. 3).

### DNA methylation comparison in 2D, 3D, and FFPE

Variations in methylation signature in CRC cells cultured in 2D and 3D models and FFPE samples were calculated (Table 2). An increase in mean methylation rate (19.1 ± 7.4) was significantly observed in the 2D model for CRC cells compared with the same cells cultured in the 3D model and FFPE sample (7.07 ± 3.1, *p* = 0.0005 and 4.41 ± 3.3, *p* < 0.0001, respectively). Interestingly, no variation was recorded between 3D cultured cells and FFPE samples.

**Table 2:** Percentage of 5-mC level in CRC cell lines growing in 2D and 3D culture models and corresponding FFPE blocks of patients with CRC cancer. Results represent mean ± standard deviation of three independent experiments (*n* = 3).

Sample	Mean 5-mC% ± SD	Sig	*p* value
2D	19.10 ± 7.38^a^	**	< 0.0001
3D	7.07 ± 3.11^b^		
FFPE	4.41 ± 3.32^b^		

FFPE: Formalin-Fixed Paraffin-Embedded; 2D: Two- Dimensional; 3D: Three- Dimensional; 5-mC: 5-methylcytosine. ** *p* < 0.01. Different letters (a and b) are significant at *p* < 0.01.

### miRNA expression analysis

**Figure 4:**
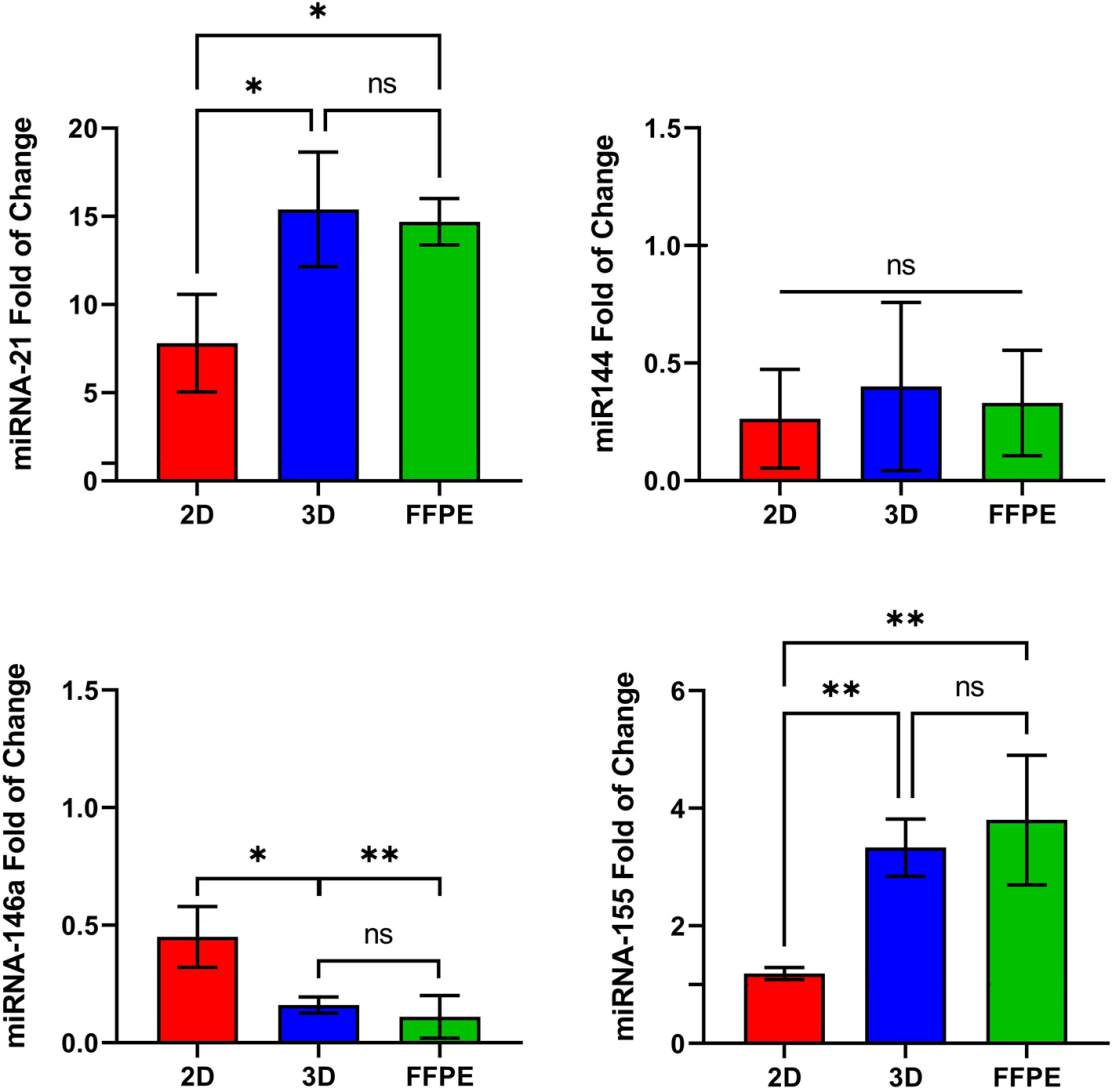
miR-21, miR-144, miR-146a, and miR-155 expression in CRC cell lines cultured in both 2D and 3D cell culture models compared with FFPE colorectal block samples. The results were normalized to RNU43 as an endogenous control. Results represent the mean ± standard deviation of three independent experiments. * *p* < 0.05; ** *p* < 0.01; NS: Non-significant; FFPE: Formalin-Fixed Paraffin-Embedded; 2D: Two- Dimensional; 3D: Three- Dimensional.

The expression of miRNA-21, miRNA-144, miRNA-146a, and miRNA-155 using RT-qPCR for CRC cell lines (pooled as one group) maintained in 2D and 3D cultures was compared with FFPE samples. miR-21 overexpressed in 2D and 3D cultures and FFPE samples. However, miR-21 in 3D culture and FFPE samples was significantly highly expressed with 1.97-fold (*p* = 0.0264) and 1.88-fold (*p* = 0.0392) compared to 2D culture model, respectively. No differences were detected between 3D cultures and FFPE blocks. miR-144 expression showed no significant variation in all tested groups. The expression profile of miRNA-146a was distinctly downregulated in 2D, 3D, and FFPE groups, with significant downregulation in 3D spheroidal cells and FFPE samples compared to 2D, monolayer culture (*p* = 0.0204 and *p* = 0.01, respectively). No variation between 3D and FFPE groups. Finally, the expression level of miRNA-155 was robustly increased in the 3D culture and FFPE group compared with tumor cells grown in the 2D model with 2.8 (*p* = 0.0033) and 3.2 (*p* < 0.0001) fold change, respectively. No significant differences were perceived between the 3D culture model and the FFPE group (Fig. 4).

### Transcriptomic analysis

**Figure 5:**
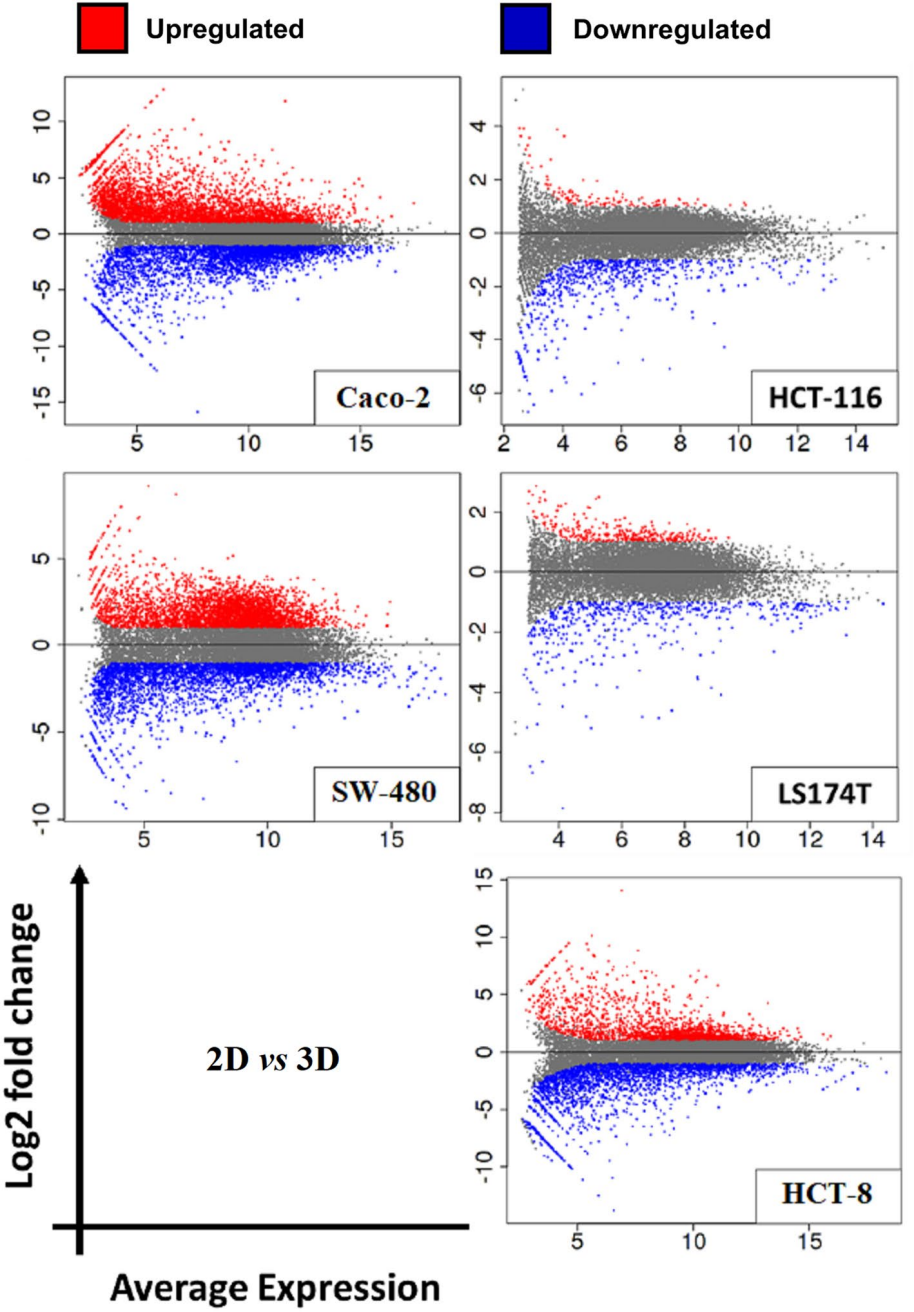
Mean-Average (MA) plots of log2 fold change versus log2 average expression between 2D and 3D culture conditions in CRC cell lines. Each dot represents a gene. Log2 fold change above 0 indicates genes with upregulated expression, while lower than 0 indicates genes with down-regulation. Red and blue dots indicate genes that significantly differ in gene expression (*p-adj* < 0.05).

Gene expression analysis was performed through RNA sequencing on samples prepared from a panel of CRC cell lines grown in 2D and 3D conditions and RNA purified from FFPE samples followed by subsequent transcriptomic comparisons between different groups. The quality of RNA isolated from FFPE samples was evaluated using DV_200_ and RIN. The median DV_200_ value was 70.8%, while the median RIN value was 2.45, both values were acceptable for the sequencing of RNA extracted from FFPE. RNA-Seq analysis (Fig. 5) revealed 7585 differential expressed genes in Caco-2 cultured in 3D condition compared with corresponding Caco-2 cells grown in 2D conditions with 4056 up-regulated genes and 3529 down-regulated genes (Log2FC < -15 or > 10 with *p-adj* < 0.05). In HCT-116, a total of 808 expressed genes were identified in comparison between 2D and 3D cultures, 127 up-regulated genes, and 681 down-regulated genes (Log2FC < -6 or > 4 with *p-adj* < 0.05). SW40 showed 7261 expressed genes with 3895 up- and 3366 down-regulated genes (Log2FC < -6 or > 4 with *p-adj* < 0.05). Only 862 differential expressed genes in 2D/3D cultures of LS174T cells were displayed with 321 up-regulated and 541 down-regulated genes (Log2FC < -8 or > 2 with *p-adj* < 0.05). Finally, the expression of varied 4684 genes was detected in 2D/3D cultures of HCT-8 cells with 2032 up-regulated and 2652 down-regulated genes (Log2FC < -6 or > 4 with *p-adj* < 0.05). Results demonstrated that gene expression profiles were highly affected by the growth conditions of 2D and 3D cultures for each cell line. Functional enrichment analysis (Supp. 4) involved all the varied expressed genes in 2D versus 3D cultures of all tumor cell lines. Among all cells, a different set of genes of different pathways exhibited distinctive characteristics of increased or decreased gene expression. For instance, in Caco-2 cells, cell growth conditions stimulated the up-regulation of gene sets associated with ion transport and different metabolic processes. In contrast, gene sets related to the mitotic cell cycle, cell division, chromosomal organization, and DNA regulation were significantly down-regulated. On the contrary, HCT-116, SW40, LS174T, and HCT-8 showed down-regulation in gene sets unrelated to proliferation processes, while genes associated with cell cycle and regulation of gene expression were upregulated.

**Figure 6:**
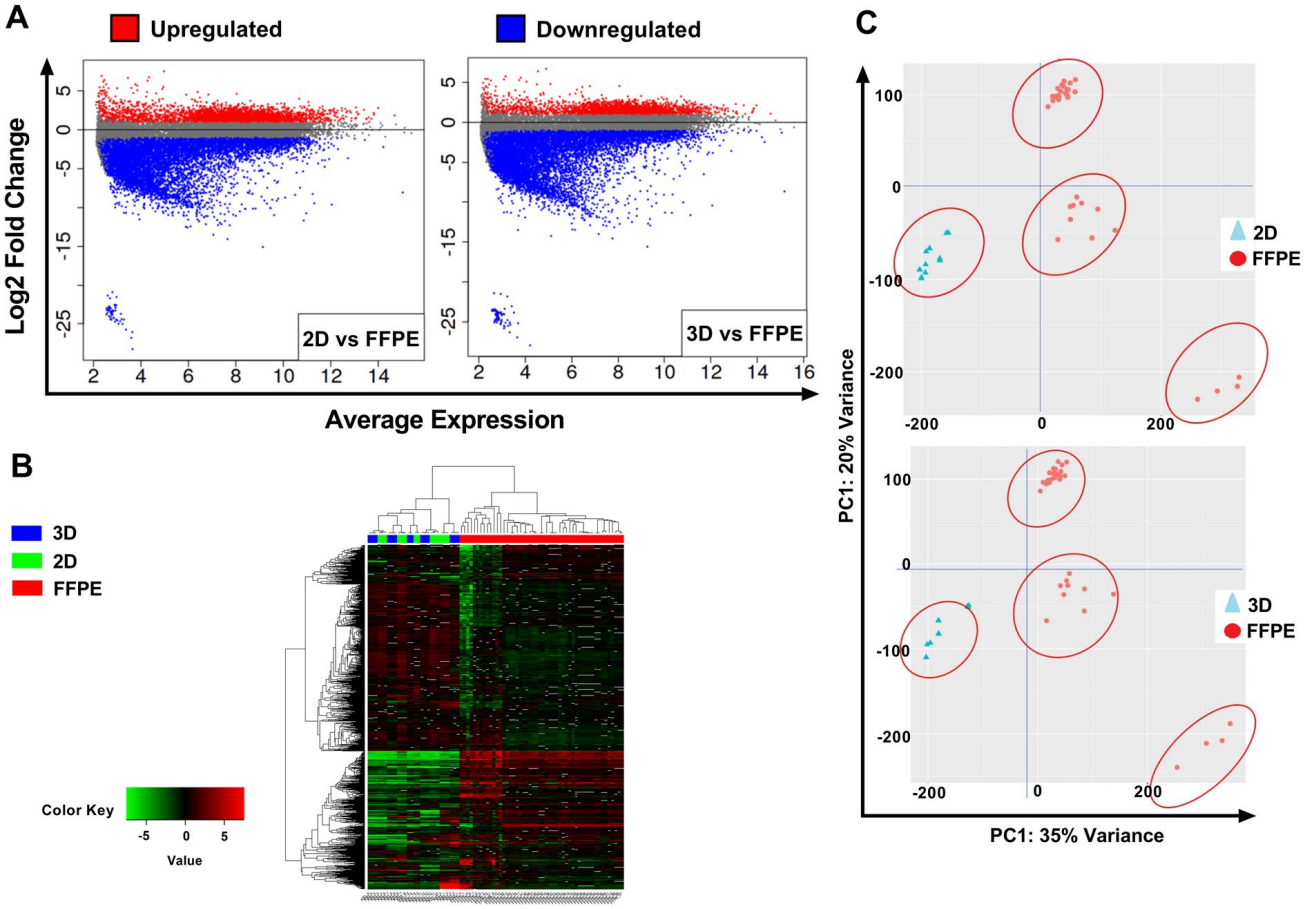
**(A)** Mean-Average (MA) plots of log2 fold change versus log2 average expression between the type of culture condition and FFPE samples. Each dot represents a gene. Log2 fold change above 0 indicates genes with upregulated expression, while lower than 0 indicates genes with down-regulation. Red and blue dots indicate genes that significantly differ in gene expression (*p-adj* < 0.05). **(B)** Hierarchical clustering heat map of log2 expression levels of most significantly (*p-adj* < 0.05) expressed genes resulting from RNA sequencing analysis of (Green) 2D cultured cells with FFPE samples (Blue) 3D cultured cells with FFPE samples. Sample data were pooled and normalized with DeSeq2, followed by H-clustering to compute the cluster dendrogram. **(C)** Principal-component analysis of sample relationship. PCA 1 is 35% and 34% variance for the relationship between 2D/3D with FFPE data. PCA2 is 20% for both. Cell lines for 2D and 3D culture conditions are clustered in one group apart from FFPE clustering. FFPE: Formalin-Fixed Paraffin-Embedded.

Further gene expression was compared between RNA-Seq results obtained from FFPE samples and the bulk results of 2D and 3D experiments for all cell lines. As expected, vast variation in gene expression resulted between FFPE and tumor cells cultured in 2D conditions, which resulted in the identification of 13,755 expressed genes with 3572 up-regulated genes and 10,183 down-regulated genes (Log2FC < -25 or > 5 with *p-adj* < 0.05). Surprisingly, totally accounting for 13,629 differentially expressed genes were identified between FFPE samples and cell lines grown in 3D culture conditions, 2726 up-regulated and 10,903 down-regulated genes (Log2FC < -25 or > 5 with *p-adj* < 0.05) (Fig. 6A). Based on unsupervised hierarchical clustering algorithm, FFPE gene expression partition in clusters significantly (*p-adj* < 0.05) different from the expression of 2D or 3D cultured cells (Fig. 6B). Principal component analysis (PCA) of variant genes re-marked such segregation (Fig. 6C).

## Discussion

Several studies mentioned various methods that can be used to grow cells in 3D culture. These methods can be divided into two main types. The first is scaffold-based 3D culture techniques, which provide a supportive surface for cellular growth. The second type is scaffold-free techniques^44^. In the proliferation assay, the low adhesion technique was adopted. It is one of the most important scaffold-free 3D cell culture techniques^45^. This method ensures that cells do not attach to the well surface and allows them to clump together and form their specific extracellular matrix. Furthermore, this method is considered a lower cost than other 3D cell culture techniques because it does not need any treatments or additives, as in the case of using scaffolding, and is easy in spheroid formation. Finally, this method allows cells to create a spheroid that can be used as a model for various laboratory experiments^46^.

Different cell lines proliferate faster in 2D cultures compared to 3D cultures^45,47^. However, other cell lines proliferated slower in the 2D cell culture system in another experiment^48^. The increase in cell proliferation rate in 2D conditions is related to the stratified structure, where more cells are exposed to nutrients and growth factors in the culture medium, making them more prone to growth at a reasonably uniform rate on a flat surface. Other factors that contribute to the changes in the proliferation rate of 2D and 3D cultures are the cell–cell and cell-extracellular matrix interaction, which can change the expression of genes and proteins associated with cell growth and proliferation^13,49^. Cell proliferation is influenced by the type of 3D matrix and the characteristics of each cell line.

Conditions in 2D cell culture allow the growth of proliferative cells in a flat regulated environment until they reach monolayer confluence. The cells in 2D culture are characterized by uniform access to nutrients and growth factors present in culture media, which allow the cells to grow faster than the cells actually grow in vivo. In addition, cellular morphological changes are observed in stretched and flattered cells in 2D cultures compared with in vivo cells^50^. On the contrary, cells in the 3D system come in close contact and spontaneously clump together, forming spheroids that lead to cell growth at several stages: proliferation, rest, apoptosis, hypoxia, and necrotic cells. This occurs due to the limited medium diffusion into cells growing with spheroids; thus, the cells accumulated in the spheroids' core enter apoptosis and die^51^. This condition mainly explains the significant reduction in tumor cells grown in a 3D model. Increasing 3D cell population in the late apoptosis phase may refer to changes in cellular requirements for nutrients resulting from the cell's active proliferation rate and dormancy^52^.

Cells in monolayer culture are more likely to receive the same amount of chemotherapeutic agent and are predominantly proliferative cells. Therefore, 3D cell culture models containing cells at various stages tend to be more resistant to treatment with chemotherapeutic agents than traditional 2D cultures, mimicking in vivo conditions in terms of the cell cycle, cell morphology, nutrient requirement, and cellular behavior^53^. Changes in transcriptional levels may be a reflection of the physiology of cell development, adjustments to the immune system, and therapeutic resistance in tumor cells^54^. For instance, a study comparing the transcription profiles of two lung cancer cell lines revealed that, when compared to a 2D culture, the 3D culture was capable of mimicking cancer cell phenotypes such as hypoxia and angiogenesis activation that were not imitated in 2D model^55^. Furthermore, clustering analysis showed that cell lines grown in 3D cell culture conditions are clustered in separate groups, while in the second group, 2D cultured cell lines were clustered (Fig. 3B).

The *ANXA1* gene encodes a membrane-bound protein binding phospholipids, often downregulated in tumors, potentially serving as a biomarker and therapeutic target^56^. *ANXA1* overexpression is linked to increased tumor hypoxia, a characteristic of tumors often replicated in 3D multicellular spheroid cultures, mimicking in vivo conditions^57^. The *CD44* gene produces a cell-surface glycoprotein crucial for cell interactions, adhesion, and motility, generating functionally diverse isoforms through complex alternative splicing, associated with tumor metastasis. In 3D cultures compared to 2D monolayers, *CD44* is notably overexpressed^58^. The *KRT18* gene encodes keratin 18, implicated in cryptogenic cirrhosis, and it's significantly upregulated in CRC tissues and cell lines^59^. Surprisingly, 3D cultures exhibit down-regulated *KRT18* expression compared to 2D models, underscoring the substantial impact of unique 3D cultural and physiological conditions on gene expression profiles. Most cell lines showed consistent *OCT4* expression levels in 2D and 3D cultures. *OCT4* encodes a critical transcription factor for embryonic development and stem cell pluripotency. Overexpression in adult tissues relates to tumorigenesis, while *OCT4* gene knockdown inhibits proliferation and viability^60^. In contrast, *SOX2OT* gene expression universally decreased in 3D cultures versus 2D models. *SOX2* overlapping transcript (*SOX2OT*), a non-coding RNA, regulates pluripotency^61^. Its downregulation is prominent in various tumors^62^. The 3D culture model simulates in vivo tumor tissue, in line with *SOX2OT* downregulation in gastric cancer tissue samples and related cell lines. Notably, *SOX2OT* levels decrease with higher cancer grades^63^.

The metabolic state, cell–cell interaction, drug resistance transporter expression, and tumor cell signaling pathways are only a few of the variables that determine how active and potent anticancer medications are against tumor cells. These factors may be different in 2D and 3D cultures. Similar to how distinct cell groupings, such as proliferative and necrotic cell populations, are a crucial component of 3D systems that reflect the heterogeneity of tumors and modify the responsiveness of tumor cells to pharmacological therapies^8^. The 5FU, Cisplatin, and Doxo resistance mechanisms with low toxicity were observed in spheroidal tumor cells grown in 3D cultures. The aggregation of tumor cells as spheroids in 3D culture models might stimulate tumor cells' natural microenvironment to mimic that in vivo tumor morphology. The formation of spheroids for CRC cells in the 3D system enables the cells within spheroids to maintain the rate of cell proliferation in minimal form. The larger the spheroid formation, the lower the proliferation rate since more quiescent cells accumulate in the center of the spheroid, resulting in increasing resistance to chemotherapy^64^. In addition, tumor cells within spheroids upregulate the synthesis of proteins involved in cell–cell and cell–matrix interactions and enhance singling pathways that participate in drug resistance^65^. In addition, cells in 2D received an equal amount of nutrients containing anticancer drugs and are predominantly proliferative cells. On the contrary, the 3D system leads to cell growth at several stages: proliferation, rest, apoptosis, hypoxia, and necrotic cells, and with limited diffusion of the medium, the spheroids tend to be more resistant to treatment with chemotherapeutic agents^13,66^.

DNA methylation rate and gene expression differ greatly between 2D, and 3D models of CRC cells. Due to the distinct microenvironment that the 3D model can provide, cells in spheroids, cell–cell, and cell–matrix contact, as well as low, stable proliferation rate, markedly influence the epigenetic state and rate of DNA methylation^67^. The results highly support that the DNA methylation profiles of tumor cell lines grown in 3D models are closer to the corresponding primary tumor samples from which cell lines originated than in 2D monolayer models^68^.

In particular, the combination of methylation inhibitors and chemotherapy and/or targeted therapy may provide helpful information on developing efficient therapeutic approaches^69^. Before utilizing methylation inhibitors as chemotherapeutic drugs in patients with solid tumors, it is crucial to evaluate their antitumor effectiveness. It is now understood that 3D tumor tissue culture systems resemble actual tumor tissue by 85%. Hence, rather than using conventional 2D models, 3D tumor tissue culture systems can be used as in vitro model systems that considerably replicate in vivo tumor construction^70^.

Both DNA methylation and miRNAs are critical players in the intricate network of epigenetic regulation. They contribute to the modulation of gene expression in response to internal and external cues, helping cells adapt to changing conditions and maintain proper functioning^71^. DNA methylation is associated with the regulation of gene expression by gene silencing, transcriptional repression, and developmental regulation^72^. Whereas microRNAs bind to complementary regions within mRNA molecules, leading to mRNA degradation, translation inhibition, fine-tuning regulation and impact to various cellular processes^73^. The dysregulation of these epigenetic mechanisms has been associated with a range of diseases, including cancer, neurological disorders, and metabolic diseases. Understanding their roles provides insights into the complex orchestration of cellular processes and opens avenues for therapeutic interventions.

The pattern of miRNAs expression in experimental 3D models, whether upregulated or downregulated, was reported to be abundantly similar to miRNAs expression of FFPE CRC samples. Such finding strongly indicates that 3D-specific miRNAs reflect the in vivo status of CRC. miR-21 plays a role in multicellular tumor spheroid formation (MCTS) and cell–cell adhesion^74^. For that reason, miR-21 expression showed similar results between 3D cell culture and FFPE samples, even though miR-21 was already overexpressed in 2D but significantly less than in 3D cell culture and FFPE samples. On the other hand, the profile of miR-144 downregulation showed no change within 2D, 3D, and FFPE samples, indicating no influence of culture type on miR-144 expression compared with tumor tissue samples. miR-144 is an anti-metastatic effector that controls cellular pathways leading to carcinogenesis. Downregulation of miR-146a is a hallmark of promoting tumor cell proliferation, inhibiting apoptosis pathways, and increasing chemotherapy drug resistance^75^. miR-146a was presented in down expression for all cell lines (grown in 2D and 3D) and FFPE samples. However, 3D models and FFPE samples shared the same pattern of regulation, indicating that the 3D model for miR-146a expression imitates in vivo functions. Regarding miR-155, it is related to the ability of cancer cells to resist chemotherapy drugs^76^. Linking to the cytotoxicity assay, the results showed that 3D culture cell lines have high resistance to different chemotherapeutic drugs than 2D cell culture, which might explain the extensive endogenous expression of miR-155 in both 3D spheroidal cells and FFPE samples.

As previously mentioned, the 3D culture provides a model that better mimics in vivo conditions than traditional 2D culture^77^. Therefore, changes in cellular behavior in vitro due to altered culture conditions certainly reflect differential gene expression. For this assumption, total RNA sequencing after culturing tumor cell lines in 2D and 3D conditions was conducted, and transcriptomic analysis using the pipeline of RNA trimming, Hisat2 RNA alignment for NGS reads, FeatureCounts for strand-specific read counting, and DeSeq2 for gene expression analysis. Intriguingly, different culture conditions for tumor cell line growth showed a variation in gene expression under 3D growth compared to 2D with a significant count of upregulated and down-regulated expression of varied gene sets. Different biological processes mainly associated with cell adhesion, cell-to-cell contact, extracellular matrix organization, metabolic processes, and to a lesser extent cell cycle processes and many other pathways and processes controlled by clusters of thousands of genes, were implicated in functional enrichment analysis (Supp. 4). Such findings strongly suggest the alteration in gene expression in favor for cellular behaviors and functions related to cell maintenance in the 3D microenvironment. On the other hand, functions like DNA repair were not directly linked to cell behavior since no change in the level of gene expression was observed^78^.

Transcriptome analysis revealed significant variations between 2D and 3D models. However, when compared to FFPE samples, both 2D and 3D models clustered together, suggesting that while the behavior of tumor cell lines differs in 2D and 3D cultures, these differences are subtler than those observed in vivo^79^. Many researchers argue that 3D models closely mimic in vivo conditions^80,81^, and preliminary experiments in this study support this notion. Nevertheless, comprehensive transcriptomic analysis via RNA sequencing still identified gene expression differences between 2D and 3D cultures. However, these distinctions appear inconsequential when compared to FFPE samples, implying that they do not significantly contribute to overall gene expression variation. Despite 3D models not perfectly mirroring in vivo conditions, evidence from various assays suggests they closely resemble them. Thus, employing 3D culture for tailored cancer therapies based on genetic mutations, altered pathways, and molecular patterns is pivotal for advanced personalized treatment.

## Conclusion

According to the two null argumentative hypotheses that were posed at the beginning of this study, we can conclude the following. A significant variation between CRC cell line cultured in 2D and 3D models existed which is related to proliferation, expression of genes associated with tumorgenicity, cytotoxicity, pattern of methylation and miRNA expression. RNA sequencing, gene expression analysis, and functional enrichment analysis convincingly demonstrated that 2D and 3D models were significantly different, the first hypothesis was disapproved. The second hypothesis is still controversial, by comparing 2D/3D cultures with FFPE samples, both 2D and 3D were clustered in one group, whereas FFPE clustered in a distinct group.

### Supplementary Information

Supplementary Information 1.

Supplementary Information 2.

Supplementary Information 3.

Supplementary Information 4.

## Data Availability

All data and materials supporting the conclusions of this research are available in the article including supplementary files. The datasets generated and/or analysed during the current study are available in NCBI—Bioproject repository, PRJNA1020799.
