# Supplementary material for: Comparative analysis between 2D and 3D colorectal cancer culture models for insights into cellular morphological and transcriptomic variations
Source: Sci Rep. 2023 Oct 26;13:18380. doi: 10.1038/s41598-023-45144-w (PMC10603139; doi:10.1038/s41598-023-45144-w)
Supplement: Supplementary file 1 — Supplementary Information 1. [file 41598_2023_45144_MOESM1_ESM.pdf]

Supp. 1: Mean (±SD) percentage of cell viability of Caco-2, HCT-116, SW-480, LS147T, and HCT-8 cell lines growing in 2D and 3D cell culture systems at 37ºC, 5% CO_2 for seven days, *n* = 6.

Time (Day)	Caco-2 Viability % (Mean±SD)			HCT-116 Viability % (Mean±SD)		
	2D	3D	*p* value	2D	3D	*p* value
1	100	100	------	100	100	------
2	120.8±5.5	153.7±8.1	<0.0001**	141.3±6.8	137.8±6.1	0.7718NS
3	81.3±3.7	174.9±9.2	<0.0001**	95.1±4.5	156.7±7.0	<0.0001**
4	50.2±2.3	190.6±10.01	<0.0001**	58.8±2.8	155.1±6.8	<0.0001**
5	22.8±1.04	185.7±9.9	<0.0001**	26.7±1.3	140.2±6.2	<0.0001**
6	7.8±0.32	152.9±8.02	<0.0001**	8.5±0.4	115.5±5.1	<0.0001**
7	1.3±0.06	136.5±1.2	<0.0001**	1.5±0.1	103.1±4.6	<0.0001**
**Time (Day)**	**SW480 Viability % (Mean±SD)**			**LS174T Viability % (Mean±SD)**		
	**2D**	**3D**	***p* value**	**2D**	**3D**	***p* value**
1	100	100	------	100	100	------
2	90.6±8.2	106.2±5.3	<0.0001**	123.6±10.3	110.4±2.7	<0.0001**
3	60.9±5.5	110.2±5.5	<0.0001**	83.2±6.9	131.03±3.2	<0.0001**
4	37.7±3.4	125.4±6.2	<0.0001**	51.4±4.3	123.8±3.0	<0.0001**
5	17.1±1.6	108.3±5.4	<0.0001**	23.4±1.9	114.4±2.8	<0.0001**
6	5.5±0.5	84.9±4.2	<0.0001**	7.4±0.6	102.3±2.5	<0.0001**
7	0.98±0.1	67.3±3.3	<0.0001**	1.4±0.1	88.8±2.2	<0.0001**

Time (Day)	HCT-8 Viability % (Mean±SD)		
	2D	3D	*p* value
1	100	100	------
2	111.5±7.9	138.5±31.7	0.0574
3	77.9±7.7	143.9±30.2	<0.0001**
4	67.6±9.8	132.5±19.2	<0.0001**
5	47.7±6.8	131.6±18.9	<0.0001**
6	32.2±5.8	111.0±15.9	<0.0001**
7	17.8±3.9	102.8±30.7	<0.0001**

**: $p \leq 0.01$, NS: Non-Significant, SD: Standard Deviation, 2D: Two-Dimensional, 3D: Three-Dimensional.
