# Supplementary material for: Comparative analysis between 2D and 3D colorectal cancer culture models for insights into cellular morphological and transcriptomic variations
Source: Sci Rep. 2023 Oct 26;13:18380. doi: 10.1038/s41598-023-45144-w (PMC10603139; doi:10.1038/s41598-023-45144-w)
Supplement: Supplementary file 3 — Supplementary Information 3. [file 41598_2023_45144_MOESM3_ESM.pdf]

Supp. 3: Mean (±SD) inhibition percentage and half maximal inhibition (IC_{50}) values for 5- fluorouracil, cisplatin and doxorubicin treated colorectal cancer cells in 2D and 3D culture models (*n* = 6).

Caco-2

Drug	Conc. µg mL^{-1}	2D Mean (±SD) Inhibition (%)			IC_{50} 2D	3D Mean (±SD) Inhibition (%)			IC_{50} 3D	Sig.
**5FU**	500	63.5	±	2.3	**18.2**	53.5	±	1.3	**100.6**	**
	250	61.5	±	1.4		49.5	±	1.3		**
	125	59.7	±	2.4		41.5	±	1.6		**
	62.5	55.5	±	1.9		6.6	±	1.2		**
	33.3	39.9	±	1.7		4.5	±	1.8		**
	16.6	10.0	±	1.3		3.9	±	1.2		**
**Cisplatin**	1000	89.8	±	2.2	**107.0**	81.8	±	1.9	**315.9**	**
	500	90.3	±	2.4		74.7	±	2.5		**
	250	82.2	±	2.5		36.8	±	1.7		**
	125	52.1	±	2.7		24.2	±	1.7		**
	62.5	31.6	±	2.1		24.8	±	1.8		**
	33.3	22.8	±	1.9		25.4	±	1.2		NS
	16.6	5.0	±	1.5		4.7	±	1.1		NS
**Doxorubicin**	250	77.2	±	1.3	**27.1**	57.3	±	1.5	**47.6**	**
	125	76.4	±	1.5		54.1	±	1.2		**
	62.5	73.7	±	1.6		43.7	±	1.9		**
	31.3	57.6	±	1.5		11.3	±	1.2		**
	15.6	8.7	±	1.5		4.5	±	1.7		*
	7.8	7.6	±	1.6		3.3	±	1.4		*

HCT-116

Drug	Conc. µg mL^{-1}	2D Mean (±SD) Inhibition (%)			IC_{50} 2D	3D Mean (±SD) Inhibition (%)			IC_{50} 3D	Sig.
**5FU**	500	90.3	±	2.2	**69.7**	79.0	±	2.2	**207.9**	**
	250	89.8	±	2.2		60.4	±	0.9		**
	125	79.2	±	2.5		26.6	±	1.4		**
	62.5	51.1	±	1.9		19.7	±	2.3		**
	33.3	30.6	±	1.9		18.9	±	1.8		**
	16.6	22.8	±	3.2		18.4	±	1.8		NS
**Cisplatin**	1000	82.7	±	1.7	**115.4**	74.7	±	1.9	**319.5**	**
	500	83.2	±	1.5		67.6	±	2.4		**
	250	75.0	±	2.3		29.6	±	1.9		**

	125	44.0 ± 1.8		17.1 ± 1.7		**
	62.5	23.5 ± 1.3		17.7 ± 1.5		**
	33.3	15.7 ± 1.4		18.3 ± 1.9		NS
	16.6	1.0 ± 1.9		2.9 ± 1.8		NS
**Doxorubicin**	250	96.0 ± 2.1	**34.5**	88.0 ± 1.8	**82.9**	**
	125	96.5 ± 1.9		80.9 ± 1.3		**
	62.5	88.4 ± 1.7		43.0 ± 1.9		**
	31.3	57.3 ± 1.4		30.4 ± 2.7		**
	15.6	36.8 ± 1.9		31.0 ± 1.9		**
	7.8	29.0 ± 2.4		31.6 ± 1.8		NS

SW-480

**Drug**	**Conc. μg mL^{-1}**	**2D Mean (±SD) Inhibition (%)**	**IC_{50} 2D**	**3D Mean (±SD) Inhibition (%)**	**IC_{50} 3D**	**Sig.**
**5FU**	500.0	80.2 ± 1.7	**51.7**	56.1 ± 1.9	**88.7**	**
	250.0	79.3 ± 2.1		50.0 ± 2.1		**
	125.0	71.5 ± 1.9		40.2 ± 2.0		**
	62.5	53.6 ± 2.2		28.8 ± 1.7		**
	33.3	36.1 ± 0.9		17.7 ± 1.6		**
	16.6	21.8 ± 1.3		13.0 ± 1.5		**
**Cisplatin**	1000.0	56.3 ± 2.2	**53.8**	50.0 ± 1.4	**199.3**	**
	500.0	56.3 ± 1.8		45.0 ± 1.7		**
	250.0	56.3 ± 1.7		38.0 ± 2.0		**
	125.0	51.0 ± 1.9		10.0 ± 2.3		**
	62.5	36.4 ± 2.1		6.5 ± 1.9		**
	33.3	6.5 ± 1.9		6.5 ± 1.6		NS
	16.6	0.7 ± 1.7		6.4 ± 1.5		*
**Doxorubicin**	500.0	79.6 ± 1.4	**55.3**	59.8 ± 2.6	**95.8**	**
	250.0	79.8 ± 1.9		56.6 ± 1.6		**
	125.0	77.1 ± 2.4		46.1 ± 1.9		**
	62.5	60.0 ± 2.0		13.7 ± 2.1		**
	33.3	11.1 ± 1.0		6.9 ± 2.3		*
	16.6	10.0 ± 1.9		5.7 ± 1.8		*

LS174T

Drug	Conc. µg mL^{-1}	2D Mean (±SD) Inhibition (%)			IC_{50} 2D	3D Mean (±SD) Inhibition (%)			IC_{50} 3D	Sig.
**5FU**	500	76.0	±	1.4	**69.7**	58.3	±	1.4	**207.9**	**
	250	75.1	±	1.7		56.5	±	1.6		**
	125	67.2	±	2.1		51.3	±	1.9		**
	62.5	49.4	±	1.6		32.4	±	2.4		**
	33.3	31.8	±	1.5		12.2	±	1.2		**
	16.6	17.6	±	1.3		9.8	±	1.6		**
**Cisplatin**	1000	60.0	±	1.7	**115.4**	50.0	±	2.5	**319.5**	**
	500	58.0	±	1.4		46.0	±	2.6		**
	250	56.3	±	1.9		38.0	±	1.5		**
	125	52.0	±	1.2		3.1	±	1.7		**
	62.5	36.4	±	2.1		1.0	±	2.1		**
	33.3	6.5	±	2.2		0.5	±	1.3		**
	16.6	0.7	±	1.4		0.0	±	1.6		NS
**Doxorubicin**	250	85.1	±	1.5	**34.5**	77.1	±	1.5	**82.9**	**
	125	85.6	±	1.9		70.0	±	1.3		**
	62.5	77.5	±	1.5		32.1	±	1.6		**
	31.3	46.5	±	1.3		19.5	±	1.4		**
	15.6	25.9	±	2.2		20.1	±	1.8		**
	7.8	18.1	±	1.6		20.7	±	1.2		NS

HCT-8

Drug	Conc. µg mL^{-1}	2D Mean (±SD) Inhibition (%)			IC_{50} 2D	3D Mean (±SD) Inhibition (%)			IC_{50} 3D	Sig.
**5FU**	500	66.3	±	2.3	**88.59**	61.1	±	1.3	**59.31**	**
	250	61.7	±	1.4		59.2	±	1.3		NS
	125	46.9	±	2.4		54.0	±	1.6		**
	62.5	22.9	±	1.9		38.2	±	1.2		**
	33.3	10.3	±	1.7		18.0	±	1.8		**
	16.6	3.57	±	1.3		12.5	±	1.2		**
**Cisplatin**	1000	62.5	±	2.2	**54.44**	59.2	±	1.9	**172.70**	NS
	500	60.5	±	2.4		55.2	±	2.5		*
	250	58.7	±	2.5		47.2	±	1.7		**
	125	54.5	±	2.7		22.4	±	1.7		**
	62.5	38.9	±	2.1		13.2	±	1.8		**
	33.3	8.98	±	1.9		9.7	±	1.2		NS

	16.6	3.12	±	1.5		9.2	±	1.1		**
**Doxorubicin**	250	83.4	±	1.3	**34.53**	79.9	±	1.5	**107.40**	*
	125	83.9	±	1.5		59.4	±	1.2		**
	62.5	75.8	±	1.6		25.5	±	1.9		**
	31.3	44.8	±	1.5		18.2	±	1.2		**
	15.6	24.2	±	1.5		18.6	±	1.7		**
	7.8	16.4	±	1.6		18.9	±	1.4		NS
